# Effects of trimetazidine on heart failure with reduced ejection fraction and associated clinical outcomes: a systematic review and meta-analysis

**DOI:** 10.1136/openhrt-2023-002579

**Published:** 2024-05-08

**Authors:** Soufiane Nassiri, Arno A Van de Bovenkamp, Sharon Remmelzwaal, Olimpia Sorea, Frances de Man, M Louis Handoko

**Affiliations:** 1 Cardiology, Amsterdam University Medical Centres, Amsterdam, Netherlands; 2 Amsterdam Cardiovascular Sciences, Amsterdam, Netherlands; 3 Epidemiology & Biostatistics, Amsterdam University Medical Centres, Amsterdam, Netherlands; 4 Pulmonary Medicine, Amsterdam University Medical Centres, Amsterdam, Netherlands

**Keywords:** Heart Failure, Diastolic, Heart Failure, Systolic, Pharmacology, Meta-Analysis

## Abstract

**Background:**

Despite maximal treatment, heart failure (HF) remains a major clinical challenge. Besides neurohormonal overactivation, myocardial energy homoeostasis is also impaired in HF. Trimetazidine has the potential to restore myocardial energy status by inhibiting fatty acid oxidation, concomitantly enhancing glucose oxidation. Trimetazidine is an interesting adjunct treatment, for it is safe, easy to use and comes at a low cost.

**Objective:**

We conducted a systematic review to evaluate all available clinical evidence on trimetazidine in HF. We searched Medline/PubMed, Embase, Cochrane CENTRAL and ClinicalTrials.gov to identify relevant studies.

**Methods:**

Out of 213 records, we included 28 studies in the meta-analysis (containing 2552 unique patients), which almost exclusively randomised patients with HF with reduced ejection fraction (HFrEF). The studies were relatively small (median study size: N=58) and of short duration (mean follow-up: 6 months), with the majority (68%) being open label.

**Results:**

Trimetazidine in HFrEF was found to significantly reduce cardiovascular mortality (OR 0.33, 95% CI 0.21 to 0.53) and HF hospitalisations (OR 0.42, 95% CI 0.29 to 0.60). In addition, trimetazidine improved (New York Heart Association) functional class (mean difference: −0.44 (95% CI −0.49 to −0.39), 6 min walk distance (mean difference: +109 m (95% CI 105 to 114 m) and quality of life (standardised mean difference: +0.52 (95% CI 0.32 to 0.71). A similar pattern of effects was observed for both ischaemic and non-ischaemic cardiomyopathy.

**Conclusions:**

Current evidence supports the potential role of trimetazidine in HFrEF, but this is based on multiple smaller trials of varying quality in study design. We recommend a large pragmatic randomised clinical trial to establish the definitive role of trimetazidine in the management of HFrEF.

**WHAT IS ALREADY KNOWN ON THIS TOPIC**

- Previous systematic reviews on trimetazidine in heart failure may suggest clinical benefits. However, these reviews may be dated and/or have only evaluated only a particular subgroup or clinical endpoint. Furthermore, non-English literature has mostly been ignored.

**WHAT THIS STUDY ADDS**

- In this systematic review, we have included 28 studies bringing together 2–3 fold the amount of previously available clinical evidence.

**HOW THIS STUDY MIGHT AFFECT RESEARCH, PRACTICE OR POLICY**

- We observed the clinical benefits of trimetazidine in heart failure with reduced ejection fraction across all studied clinical endpoints. However, the quality of evidence was substandard. Nonetheless, there is a sufficient rationale to conduct a large randomised clinical trial to establish the role of trimetazidine in the management of heart failure.

## Introduction

Heart failure (HF) is a common, disabling and lethal condition, with significant societal implications, including high disease burden and associated medical costs.^1^ Recent analysis demonstrated that the lifetime risk of developing HF has risen to one out of four individuals^2^ and with an ageing population, the prevalence of HF is expected to rise even further. While considerable progress has been made in the management of HF with reduced ejection fraction (HFrEF) in the last decades, the residual risk remains unacceptably high for HF with preserved ejection fraction (HFpEF), which accounts for half of all new HF cases. The situation is even more pressing, as therapeutic options are limited, and therefore, novel additional treatments are necessary.^1^

Currently, medical therapy in HF is targeting neurohormonal overactivation, a compensatory mechanism to maintain haemodynamic homoeostasis in the short term, but which becomes detrimental in the long run.^3^ The energy depletion hypothesis expands on this view in HF, suggesting that the failing heart experiences an energy deficit due to impaired mitochondrial oxidative capacity.^4 5^ Research has found impaired myocardial energy status in both HFrEF and HFpEF.^6 7^ Therefore, drugs that could modify cardiac metabolism may be beneficial. Trimetazidine, a drug currently used for treatment of angina pectoris, partially inhibits mitochondrial fatty acid β-oxidation.^8^ It thereby concomitantly increases glucose oxidation, which enhances energy production in the heart. As a result, more units of ATP can be produced per mole of oxygen, improving mitochondrial efficiency.

Several small trials have suggested positive clinical effects of trimetazidine. In addition, trimetazidine is available at a low cost and has a favourable pharmacological profile.^8^ Side effects of trimetazidine, such as tremors, are easily recognised and cease after stopping its use. Its safety has been proven in the elderly, and in patients with renal impairment, down to an estimated glomerular filtration rate (eGFR) of 30 mL/min/1.73 m^2^. With simple dose adjustments, it may be used in even lower eGFR ranges.^9^ Furthermore, while the uptitration of current HF medication is frequently hindered by their haemodynamic (side)effects, no such effects exist with trimetazidine. Therefore, trimetazidine could serve as an interesting therapeutic adjunct in HF.

Previous systematic reviews have explored the use of trimetazidine in HF but have mostly ignored non-English literature thus far. In addition, the reviews may be dated and/or have only evaluated a particular subgroup or clinical endpoint.^10–15^ In this systematic review, we aim to comprehensively summarise all available clinical evidence on trimetazidine treatment in HF, focusing on (cardiovascular) mortality, HF hospitalisation, functional capacity (New York Heart Association (NYHA) functional class; 6 min walk distance, 6MWD), quality of life (QoL) and safety/tolerability.

## Methods

### Search strategy

This systematic review has been registered on PROSPERO (#386866). On 1 June 2022, the electronic literature search in PubMed/MEDLINE, Embase, Cochrane CENTRAL and ClinicalTrials.gov was finalised, following the PRISMA 2020 statement.^16^ We used the combination of (variance of) the terms: “heart failure” and “trimetazidine”; if available (MEDLINE, Embase), a database-specific filter for randomised clinical trials (RCTs) was applied (see online supplemental tables A1A–D).^17^ Finally, from the references of the selected full papers, additional papers were retrieved through a snowballing strategy.

10.1136/openhrt-2023-002579.supp1 Supplementary data

### Study selection

After the initial search, duplicates were removed, and all identified records were evaluated by independent researchers (SN, AAVdB and MLH) to determine if they met the criteria of the review question (1) RCT, (2) HF patients, (3) medical intervention with trimetazidine and (4) control group treated with a placebo or standard of care/open label), based on title and abstract alone. Any discrepancies in selection were settled through mutual discussion. Thereafter, full papers were retrieved and re-evaluated for suitability. Abstracts from conferences were excluded due to the inability to assess study quality. There were no language restrictions.

### Data extraction and risk-of-bias assessment

From the final selection, data extraction was performed by independent researchers by double data entry (OS, SR and MLH). Key features of the study design and general characteristics of the study population were collected. Clinical endpoints of interest were defined as reported by the original study. Risk of bias was assessed using the Cochrane RoB2-tool.^18^

### Meta-analysis

Statistical analysis was performed (SR and FdM) in R Statistics (V.4.3.0) using the meta-package (V.6.2–1). A p<0.05 was considered statistically significant.

For dichotomous outcomes, common (or fixed)-effects and random-effects meta-analyses were performed with the Peto method for pooling to determine differences in (cardiovascular) mortality and HF hospitalisations between treatment and control groups (results of the common-effect model are preferentially reported in the text, as low event rates were anticipated).

For continuous outcomes, common-effects and random-effects meta-analyses were performed to determine mean differences (NYHA functional class, 6MWD) or standardised mean differences because of varying measuring instruments (QoL). A high score on the Minnesota Living With Heart Failure Questionnaire (MLWHFQ) means poor QoL.^19^ Therefore, MLWHFQ scores were multiplied by −1 in order to quantitatively meta-analyse the QoL data.

To determine the heterogeneity, I^2^, tau^2^ and prediction intervals are reported for all meta-analyses.

Visual inspection by funnel plot and Egger’s test were used to identify potential publication bias.

The possible effect of baseline values of NYHA class, 6MWD, QoL, was visualised using a bubble plot with a regression line, and a meta-regression was performed, instead of a pretest and post-test meta-analysis.^20^ For the meta-regression, the different QoL scores were normalised for their maximal range, and additionally, MLWHFQ scores were reversed, as a high MLWHFQ score pertains to low QoL.

Prespecified subgroup analyses were limited to low versus some concerns/high risk of bias and ischaemic versus non-ischaemic cardiomyopathy. As a sensitivity analysis, we evaluated the effect of trimetazidine in studies that only included HFrEF patients. In the case of an obvious outlier, a leave-one-out evaluation was performed.

## Results

### Search results

**Figure 1:**
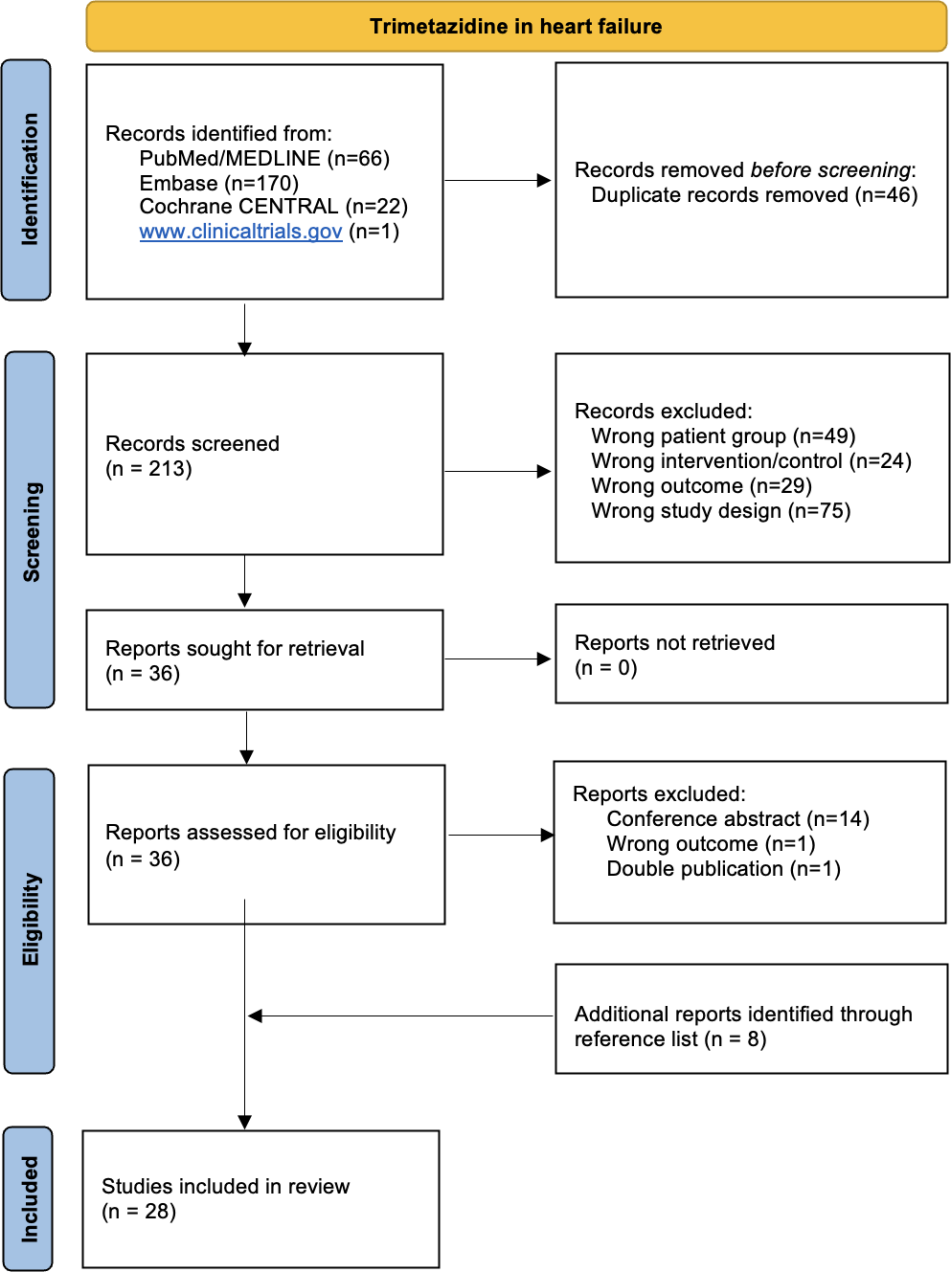
PRISMA flow diagram: 213 citations were screened and eventually 28 papers were included in the meta-analysis. PRISMA, Preferred Reporting Items for Systematic Reviews and Meta-Analyses.

From the four accessed databases, we retrieved 213 unique records. After evaluation by title and abstract, 36 records were selected for full-text evaluation (figure 1, online supplemental appendix table A2). Finally, 28 studies were selected for meta-analysis, including 2552 patients in total (table 1 (summary); online supplemental table A3).

**Table 1:** Key features on study design and general characteristics on study population of the included studies (full table in online supplement)

Study	Patient		Trimetazidine		Control	Reported outcomes of interest
First author	N (I/C)	Incl. criteria	Daily dose (mg)	Follow-up (months)	Placebo/SoC (HF-therapy)	
Bohdan^34^	22/23	HFrEF/iCMP	2×35	6	Open label (A, B, M)	M, H, FC, 6, QoL (MacNew)
van de Bovenkamp^22^	25*	HFpEF	2×35	4	Placebo (A, B, M)	M, FC, 6, QoL (KCCQ)
Bricaud^35^	9/11	HFrEF/iCMP	3×20	6	Placebo (NR)	M, FC
Brottier^36^	9/11	HFrEF/iCMP	3×20	6	Placebo (NR)	H, FC
Bubnova^23^	691/115	iCMP	–	12	Open label (A, B, M)	H, FC
Cera^37^	17/13	HFrEF/iCMP	3×20	6	Open label (A, B)	FC
Coats^21^	27/24	HCM/n-iCMP	3×20	3	Placebo (NR)	6, QoL (MLWHFQ)
Fedorova^24^	20/24	HFrEF/iCMP	3×20	6	Open label (A, B)	M, FC, QoL (MLWHFQ)
Fragasso^38^	28/27	HFrEF	3×20	12	Open label (A, B)	M, H, FC, QoL (VAS)
Fragasso^6^	12*	HFrEF	3×20	3	Open label (A, B)	FC, QoL (VAS)
Fragasso^39^	25/19	HFrEF	3×20	3	Open label (A, B)	FC, QoL (VAS)
Gunes^40^	51/36	HFrEF	3×20	3	Open label (A, B, M)	FC
Jatain^41^	50/50	HFrEF/n-iCMP	3×20	6	Open label (A, B)	M, H, FC, 6
El-Kady^42^	100/100	HFrEF/iCMP	3×20	24	Open label (A)	M
Momen^43^	55/53	HFrEF/iCMP	3×20	6	Placebo (A, B, M)	H, FC
Morozova^25^	40/42	HFrEF	2×35	3,5	Open label (A, B)	FC
Di Napoli^44^	30/31	HFrEF/iCMP	3×20	30	Open label (A, B, M)	M, H, FC, 6
Di Napoli^45^	25/25	HFrEF/iCMP	3×20	6	Placebo (A, B, M)	FC, 6
Qin^46^	41/41	n-iCMP	3×20	3	Open label (A)	FC, QoL (SF-36)
Sedova^26^	35/33	n-iCMP	2×35	1	Open label (A, B)	FC
Sisakian^47^	42/40	HFrEF/iCMP	2×35	3	Open label (A, B)	FC, 6
Sitnikova^27^	20/15	HFrEF/n-iCMP	2×35	12	Open label (A, B, M)	H, QoL (MLWHFQ)
Tuunanen^48^	12/7	HFrEF/n-iCMP	2×35	3	Open label (A, B)	H
Vasiuk^28^	28/12	HFrEF/iCMP	2×35	6	Open label (NR)	M, H, FC
Vitale^30^	23/24	HFrEF/iCMP	3×20	6	Placebo (A)	H, FC, QoL (VAS)
Wang^49^	39/40	All HF	2×35	0.25	Placebo (A, B, M)	FC, 6
Winter^50^	30/30	HFrEF/n-iCMP	2×35	6	Placebo (A, B, M)	M, H, FC, 6, QoL (MLWHFQ)
Zhang^29^	100/100	HFrEF/iCMP	3×20	1	Open label (NR)	M, 6

*Cross-over study.

B, beta-blocker; CMP/n-iCMP, ischaemic/non-ischaemic cardiomyopathy; FC, functional class; H, (heart failure) hospitalisation; HCM, hypertrophic cardiomyopathy; HF, heart failure; HFrEF/HFpEF, HF with (mildly) reduced or preserved ejection fraction; I/C, intervention/control group; KCCQ, Kansas City Cardiomyopathy Questionnaire; M, mineralocorticoid antagonist; M, mortality; MLWHFQ, Minnesota Living With Heart Failure Questionnaire; NR, not reported; QoL, quality of life ; SoC, standard of care ; VAS, Visual Analogue Scale.

Overall, the included studies were relatively small (median: N=53), with only 5 studies (18%) including 100 or more patients. Follow-up was short (median: 6 months), with only five studies (18%) having a minimal follow-up of 1 year. Most studies (79%) only included patients with HFrEF (median left ventricular ejection fraction or LVEF: 33%). A total of 14 studies included only patients with ischaemic cardiomyopathy (50%; 1753 patients) while 6 studies included only patients with non-ischaemic cardiomyopathy (21%; 359 patients), and the rest was a mix of both. Additionally, one study focused on hypertrophic cardiomyopathy^21^ and another on HFpEF.^22^ Median age was 62 years, and a minority of patients were female (median female participation: 23%). Six studies (21%) were conducted in Russia and not reported in English.^23–28^ Only two studies were conducted after the latest major revision of the European Society of Cardiology (ESC) HF guidelines in 2021, which explains the limited HF-background therapy (table 1, online supplemental table A3).

Since the vast majority of evidence came from studies that included HFrEF-patients only, and because of the neutral results of the two studies that included patient with preserved LVEF,^21 22^ we decided to report on the clinical effects of trimetazidine in HFrEF in the main document (additional data regarding HFpEF and hypertrophic cardiomyopathy is provided in Online supplemental file 1).

### Quality of the studies

Risk of bias was low in 8 studies (29%), there was some concern in 6 studies (21%) and risk of bias was high in 14 studies (50%) (online supplemental figure A1–A). An important source of potential bias was the open-label study design in 19 studies (68%) (online supplemental figure A1–B).

### Effect of trimetazidine on cardiovascular mortality

**Figure 2:**
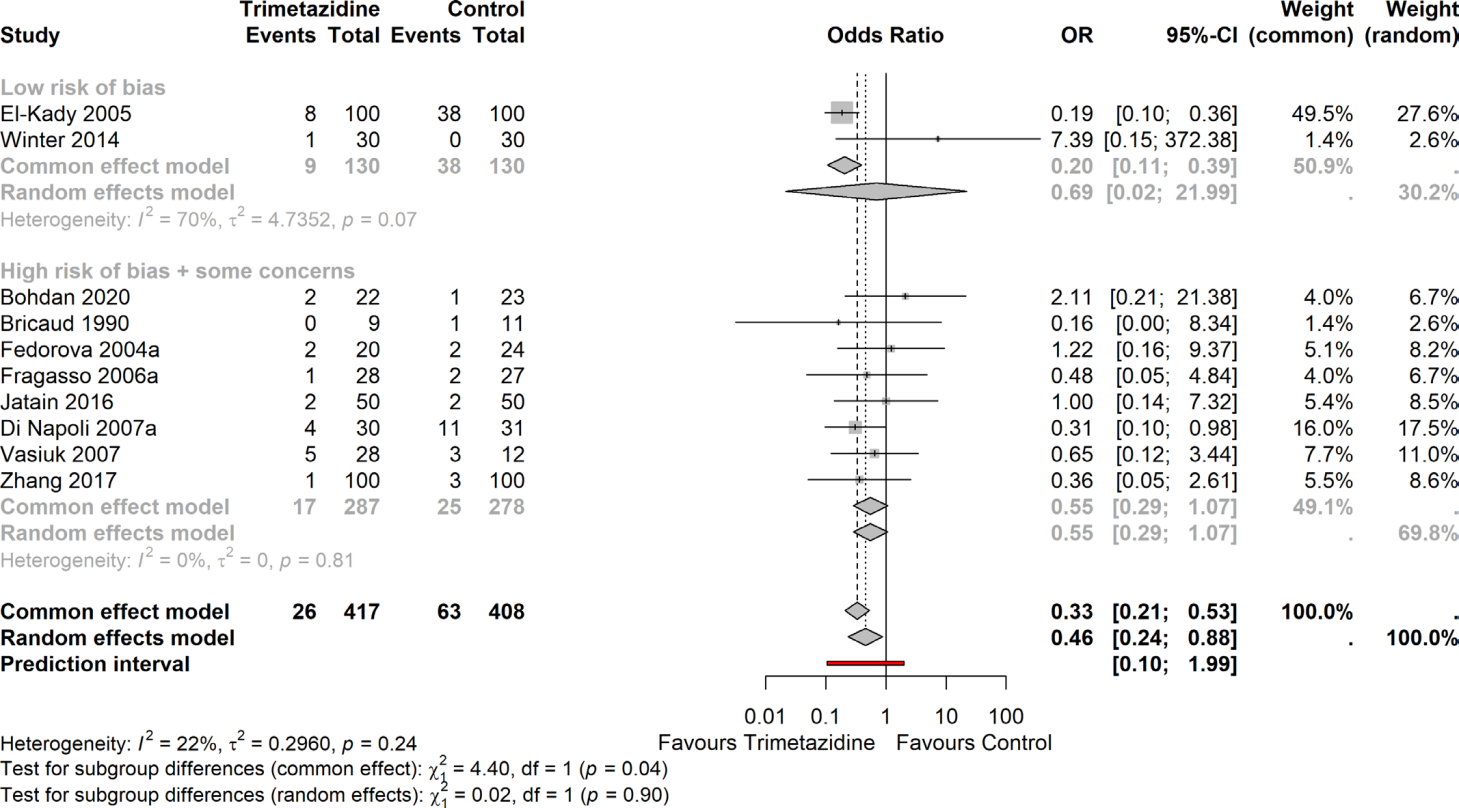
Forest plot on the effect of trimetazidine in HFrEF on (cardiovascular) mortality (10 studies), organised by low versus some concern/high risk-of-bias (p interaction=0.04). Heterogeneity I^2^: 22%; OR by common-effect or fixed-effect model: 0.33 (95% CI 0.21 to 0.53). HFrEF, heart failure with reduced ejection fraction.

10 studies in HFrEF (plus one in HFpEF) reported minimally one cardiovascular mortality event. For HFrEF, there were 89 events in total (figure 2). The OR for cardiovascular mortality in trimetazidine versus placebo/standard of care (common-effect model) was 0.33 (95% CI 0.21 to 0.53). The analysis remained unchanged if the HFpEF study was included (online supplemental figure A2–A).

Heterogeneity was limited (I^2^=22%, τ ^2^=0.30, prediction interval 0.10–1.99).

Although Egger’s test for asymmetry suggests publication bias (p<0.01), the corresponding funnel plot suggests that potential studies with larger effect sizes (at the left side of the funnel) may be missing (online supplemental figure A3–A).

There was a significant interaction between studies stratified according to low vs some concerns/high risk of bias for the common-effect model (p=0.04); the effect of trimetazidine was more pronounced in the studies with low risk of bias (OR 0.20 (95% CI 0.11 to 0.39) vs 0.55 (95% CI 0.29 to 1.07), respectively). The interaction was not observed in the random-effect model (p=0.90). Interaction in treatment effect between studies stratified according to ischaemic versus non-ischaemic cardiomyopathy, could not be assessed, as there was only one study with non-ischaemic cardiomyopathy which reported cardiovascular mortality.

### HF hospitalisation

**Figure 3:**
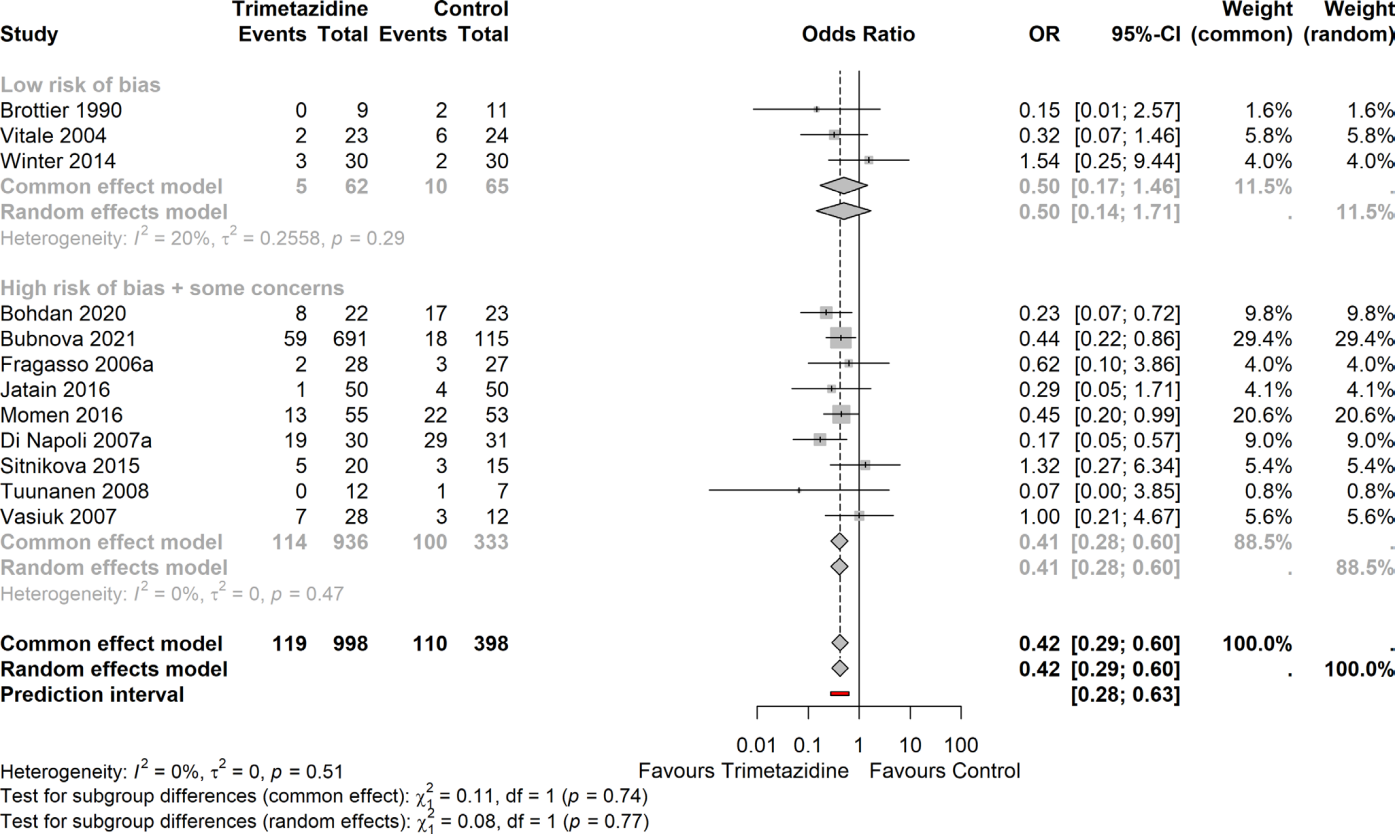
Forest plot on the effect of trimetazidine on HF-hospitalisation (12 studies), organised by low versus some concern/high risk of bias (p interaction=0.74). Heterogeneity I^2^: 0%; OR 0.42 (95% CI 0.29 to 0.60). HF, heart failure.

12 studies in HFrEF (none in HFpEF) reported minimally one HF hospitalisation event; there were 229 events in total (figure 3). The OR for HF hospitalisation in trimetazidine versus placebo/standard of care was 0.42 (95% CI 0.29 to 0.60).

Heterogeneity was limited (I^2^=0%, τ ^2^=0, prediction interval: 0.28–0.63).

There was no indication of publication bias (online supplemental figure A3–B; Egger’ test: p=0.90).

There was no interaction between studies stratified according to low versus some concerns/high risk of bias (p=0.74). There was no interaction when studies were stratified according to ischaemic versus non-ischaemic cardiomyopathy (p=0.08).

### Functional capacity

#### NYHA functional class

**Figure 4:**
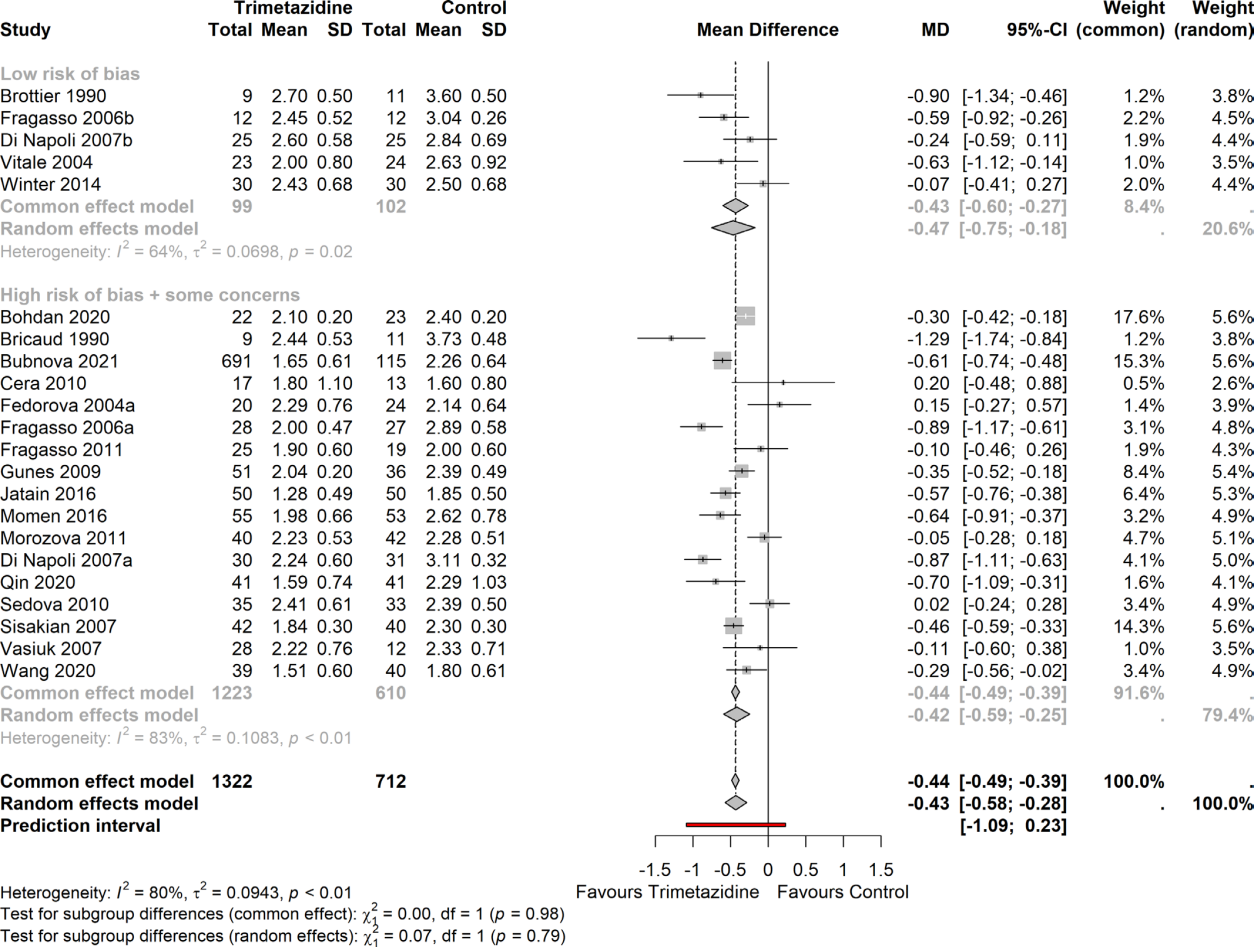
Forest plot on the effect of trimetazidine on NYHA functional class (22 studies), organised by low versus some concern/high risk of bias (p interaction=0.98). Heterogeneity I^2^: 80%; mean difference (MD) −0.44 (95% CI −0.49 to −0.39). NYHA, New York Heart Association.

22 studies in HFrEF (including 2034 patients; plus one study in HFpEF) reported on the NYHA functional class (figure 4). With trimetazidine, NYHA functional class improved (decreased) with −0.44 (95% CI −0.49 to −0.39) compared with placebo/standard of care. The overall result remained unchanged when the HFpEF study was included, despite limited NYHA improvement in HFpEF (online supplemental figure A2–B).

There was significant heterogeneity (I^2^=80%, τ ^2^=0.09, prediction interval: −1.08 to −0.25).

There was no indication of publication bias (online supplemental figure A3–C; Egger’s test: p=0.90).

Baseline values in NYHA functional class were negatively and weakly correlated with treatment effect. In other words, the treatment effect was blunted with a higher baseline NYHA functional class. However, baseline values did not explain the (between-studies) heterogeneity. There was no interaction between studies stratified according to low versus some concerns/high risk of bias (p=0.98). However, there was a significant interaction when studies were stratified according to ischaemic versus non-ischaemic cardiomyopathy (p<0.01). The effect of non-ischaemic cardiomyopathy on change in NYHA functional class was blunted, although the improvement in NYHA functional class remained significant (standardised mean difference: −0.27 (95% CI −0.39 to −0.14) vs −0.48 (95% CI −0.55 to −0.42), respectively). The interaction was not observed in a random-effects model (p=0.21).

### 6 min walking distance

**Figure 5:**
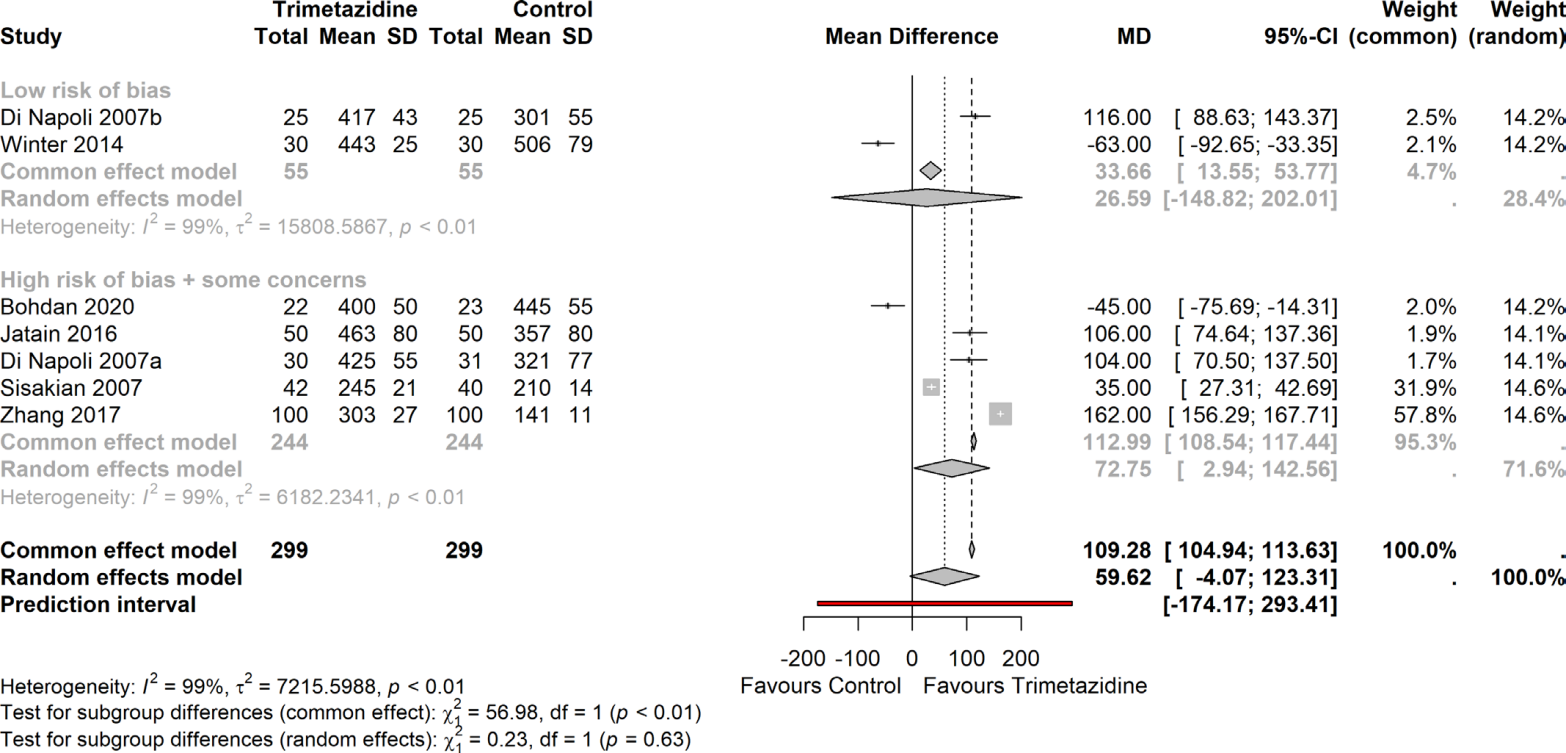
Forest plot on the effect of trimetazidine on 6 min walk distance (seven studies), organised by low versus some concern/high risk of bias (p interaction <0.01). Heterogeneity I^2^: 99%; mean difference (MD) +109 m (95% CI 105 to 114 m).

Seven studies in HFrEF (including 598 patients; plus one study in HFpEF and one in hypertrophic cardiomyopathy) reported on 6MWD (figure 5). With trimetazidine, 6MWD improved (increased) with +109 m (95% CI 105 to 114 m) compared with placebo/standard of care. The overall result remained unchanged if the studies in HFpEF and hypertrophic cardiomyopathy were included, despite limited 6MWD improvement in HFpEF (online supplemental figure A2–C).

There was significant heterogeneity (I^2^=99%, τ ^2^=7215, prediction interval: −174 to 293 m). 6MWD was less pronounced when a random-effects model was used (+60 m, 95% CI −4 to 123 m).

There was no indication of publication bias (online supplemental figure A3–D; Egger’s test: p=0.30).

Baseline values in 6MWD were negatively and weakly correlated with the treatment effect. Correction for (trimetazidine) baseline values in 6MWD explained only 24 of 99% of the (between-studies) heterogeneity. A significant interaction was observed between studies stratified according to low versus some concerns/high risk of bias for the common-effect model (p<0.01); the effect of trimetazidine was less pronounced in the studies with low risk of bias, although still significant (mean difference: +34 m (95% CI 14 to 54 m) vs +113 m, (95% CI 109 to 117 m), respectively). However, the interaction was not observed when random-effects model was used (p=0.63).

When the outlier Zhang was left out,^29^ change in 6MWD was +37 m (95% CI 30 to 44 m), but heterogeneity remained high (I^2^=96%). Interaction in treatment effect between studies stratified according to ischaemic versus non-ischaemic cardiomyopathy could not be assessed, as there was only one study with non-ischaemic cardiomyopathy that reported 6MWD.

### Quality of life

**Figure 6:**
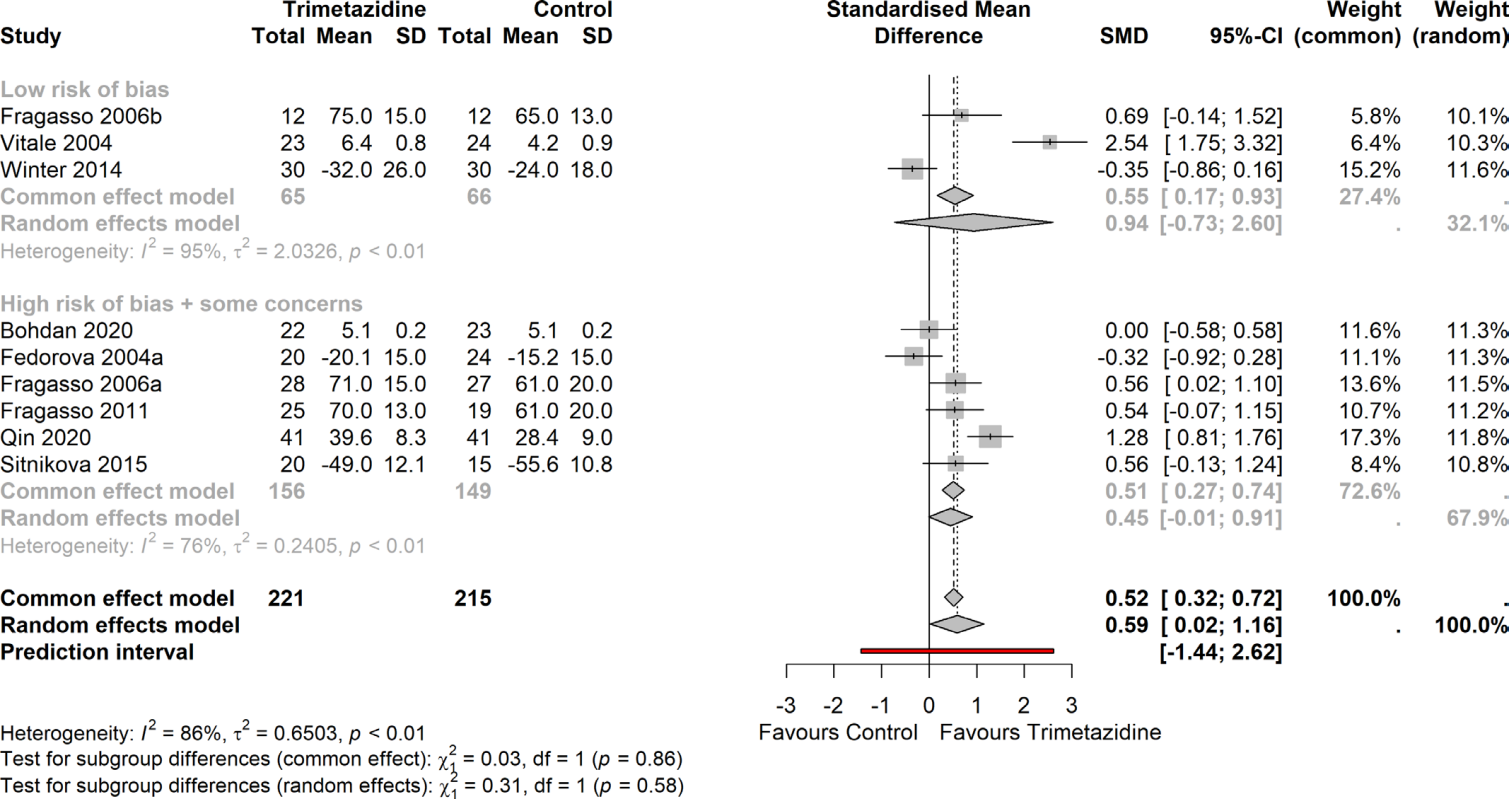
Forest plot on the effect of trimetazidine on quality of life (nine studies), organised by low versus some concern/high risk of bias (p interaction=0.86). Heterogeneity I^2^: 86%; standardised mean difference (SMD) (z-score) +0.52 (95% CI 0.32 to 0.71).

Nine studies in HFrEF (including 436 patients; plus one study in HFpEF and one in hypertrophic cardiomyopathy) reported on QoL (figure 6). Three studies used MLWHFQ, four used Visual Analogue Scale (VAS); MacNew and 36-Item Short Form Health Survey (SF-36) were each used in one study. With trimetazidine, the Z-score (standardised mean difference) improved (increased) with +0.52 (95% CI 0.32 to 0.71) compared with placebo/standard of care. The overall result remained unchanged if the studies in HFpEF and hypertrophic cardiomyopathy were included, despite limited QoL improvement in HFpEF (online supplemental figure A2–D).

There was significant heterogeneity (I^2^=86%, τ ^2^=0.65, prediction interval: −1.44 to 2.62).

There was no indication of publication bias (online supplemental figure A3–E; Egger’s test: p=0.40).

Baseline values in QoL were negatively correlated with treatment effect. Correction for (trimetazidine) baseline values in QoL explained 54 of 86% of the heterogeneity. When the outlier Vitale 2004^30^ was left out, the change in QoL was+0.38 (95% CI 0.17 to 0.58), but heterogeneity remained high (I^2^=77%). There was no interaction between studies stratified according to low versus some concerns/high risk of bias (p=0.86). There was no interaction between studies stratified according to ischaemic versus non-ischaemic cardiomyopathy (p=0.72).

### Safety/tolerability

22 studies (n=2199) mentioned the occurrence of adverse events. In these studies, no subjects prematurely discontinued trimetazidine due to side effects (table 1). The OR for the occurrence of adverse events in trimetazidine versus placebo/standard of care (common effect model) was 0.08 (95% CI 0.06 to 0.13), although heterogeneity was significant (I^2^=93%; τ ^2^=3.48).

## Discussion

To our knowledge, this is the most comprehensive systematic review of the effect of trimetazidine in HF on clinically relevant endpoints. Almost all studies were conducted in patients with HFrEF. We found that trimetazidine in HFrEF was well tolerated and associated with a significant reduction in cardiovascular mortality, HF hospitalisations and improvement in NYHA functional class, 6MWD and QoL. Trimetazidine was beneficial in both ischaemic and non-ischaemic cardiomyopathy patients. The clinical effect of trimetazidine in HFpEF was neutral.

### Comprehensiveness of the systematic review

In the past, systematic reviews have examined the effects of trimetazidine in HF (online supplemental table A4).^10–15^ However, there was a focus on surrogate endpoints (eg, LVEF or HF biomarkers/N-terminal pro b-type natriuretic peptide or NT-proBNP), and at most 11 studies were included focusing on clinically relevant endpoints. With the 28 studies included in this systematic review, we brought together 2–3 fold the amount of previously available clinical evidence (online supplemental tables A2–A4), with 15 studies not included prior systematic reviews. This was in part the result of the inclusion of non-English literature and newer studies (including a new study on HFpEF). As a result, the robustness of findings improved on all studied clinical endpoints.

With the exception of two, all studies focused on the effect of trimetazidine in HFrEF. On top of that, the two non-HFrEF studies were neutral.^21 31^ As individual participant data of the selected studies were not available, and granular information on patient characteristics (eg, age, sex, diabetes, chronic kidney function, obesity) was lacking, some of the predefined subgroup analyses were not feasible. Therefore, it is prudent to draw conclusions with these limitations in mind.

### Weighing the available evidence in HFrEF

It is reassuring that all five clinical parameters point in the same direction, suggesting a clinical benefit of trimetazidine in HFrEF. Nonetheless, there are some critical notes to make.

First of all, the conclusions are based on a collection of small studies with a short follow-up, mostly open label in design. Guideline recommendations for medical interventions in HF are typically based on the findings of more than one large RCT that recruited a couple of thousand patients with a follow-up of multiple years.^1^

It is important to note that background HF therapy used in the majority of the included studies was limited to mostly beta-blockers and ACE inhibitors only. The actual effect of trimetazidine may be smaller on top of ‘modern’ background HF-therapy.

A recent mechanistic study challenged the importance of glucose oxidation over the oxidation of fatty acids.^32^ Watson *et al* demonstrated that myocardial energetics in patients with non-ischaemic HFrEF were better with intralipid infusion compared with glucose/insulin infusions. Another mechanistic study has shown that there is a substantial difference in myocardial fuel (in)flexibility in HFpEF versus HFrEF,^33^ which may explain the larger clinical effect of trimetazidine in HFrEF than HFpEF.

Taken together, while the trial observations seem promising, it would still be far too early to routinely use trimetazidine as adjunct HF treatment in clinical practice. Furthermore, the effect sizes are most likely grossly overestimated. An HR of ~ 0.8 can already be clinically relevant and may be more realistic than the currently estimated OR of 0.33 on cardiovascular mortality, among others. Also, the heterogeneity in the meta-analyses of all clinical endpoints of interest, except for HF hospitalisations, was large to very large. Negative correlations between baseline values and effect size may suggest a ‘ceiling effect’ (minor effect in less sicker patients); however, differences in baseline values only partially explain the heterogeneity. Summation of heterogeneous study results comes with higher uncertainty. Hence, although we are inclined to concur with the consistent directional effect of trimetazidine on the different clinical endpoints, we should approach the observed effect sizes from these analyses with caution and even scepticism.

We did not find evidence of publication bias. The asymmetry in the funnel plot on mortality is not concerning, as it suggests that studies with even more pronounced treatment effects may be missing. Similarly, separating studies with low versus some concern/high risk of bias did not affect the overall conclusions. If an interaction was observed, the effect of trimetazidine was even more pronounced in the studies with low risk of bias (ie, mortality) or still remained significant (ie, 6MWD). Similar patterns were observed when separating studies into ischaemic versus non-ischaemic cardiomyopathy similar patterns were observed. It must be noted that with the low number of studies on non-ischaemic cardiomyopathy, interaction could not formally be tested for mortality and 6MWD, and the effect of trimetazidine may be blunted for the NYHA functional class (although still significantly improved). Leave-one-out analysis in case of outliers (6MWD, QoL) also did not alter results. We conclude that the findings are sufficiently robust after conducting several sensitivity analyses.

## Conclusion

Trimetazidine might offer clinical benefits in HFrEF, both in ischaemic and non-ischaemic cardiomyopathy, while data for HFpEF and hypertrophic cardiomyopathy are limited. However, this observation is based on multiple small clinical trials of mostly open-label studies. Therefore, we cannot recommend routine use of trimetazidine yet. Given trimetazidine’s favourable pharmacological profile and relatively low cost, we suggest a large pragmatic (all-comer) phase III RCT in HFrEF to evaluate whether there is a role for trimetazidine in the medical management of HF.

## Data Availability

Upon reasonable request datasets generated for this systematic review can be shared.
