# Supplementary material for: Effects of trimetazidine on heart failure with reduced ejection fraction and associated clinical outcomes: a systematic review and meta-analysis
Source: Open Heart. 2024 May 8;11(1):e002579. doi: 10.1136/openhrt-2023-002579 (PMC11086535; doi:10.1136/openhrt-2023-002579)
Supplement: Supplementary data [file openhrt-2023-002579supp001.pdf]

SUPPLEMENTAL MATERIAL

The effect of trimetazidine in heart failure with reduced ejection fraction and associated clinical outcomes: a systematic review

Soufiane Nassiri*, Arno A. van de Bovenkamp*, Sharon Remmelzwaal, Olimpia Sorea, Frances S. de Man, M. Louis Handoko

Detailed search strategy

Via OVID, PubMed/MEDLINE and Embase were assessed; Cochrane CENTRAL (which includes EudraCT) and www.clinicaltrials.gov were assessed directly. Using the "PICOTS"-structure, the general search strategy was:

Patient	heart failure
Intervention	trimetazidine
Comparator	
Outcome	
Timing	
Study design/Setting	randomised clinical trial (filter)

Only a few key search terms were used on purpose, to maximise sensitivity. Specific input for the individual databases is given below (Tables A1-4).

Supplemental tables:

Table A1-A

Search strategy OVID PubMed/MEDLINE

	Input	Hits	Comments
1	heart failure/ or heart failure, diastolic/ or heart failure, systolic/	136127	
2	Cardiac Failure.mp.	12574	
3	Heart Decompensation.mp.	133	
4	Myocardial Failure.mp.	774	
5	1 or 2 or 3 or 4	145109	1-4: different search terms for heart failure
6	trimetazidine.mp. or Trimetazidine/ or vastarel/	1195	
7	randomized controlled trial.pt.	566020	
8	controlled clinical trial.pt.	94836	
9	randomized.ab.	558426	
10	placebo.ab.	227506	
11	drug therapy.fs.	2480071	
12	randomly.ab.	380633	
13	trial.ab.	596535	
14	groups.ab.	2340038	
15	7 or 8 or 9 or 10 or 11 or 12 or 13 or 14	5329840	
16	exp animals/ not humans.sh.	4997861	
17	15 not 16	4638043	7-17: Cochrane MEDLINE RCT filter sensitivity-maximizing version (2008 revision)(18)
18	5 and 6 and 17	66	Final dataset

Table A1-B

Search strategy for Embase

	Input	Hits	Comments
1	heart failure/ or acute heart failure/ or cardiogenic shock/ or cardiopulmonary insufficiency/ or cardiorenal syndrome/ or congestive heart failure/ or diastolic dysfunction/ or forward heart failure/ or heart outflow tract obstruction/ or heart ventricle failure/ or heart ventricle overload/ or high output heart failure/ or systolic dysfunction/	417477	
2	heart failure/ or heart failure with preserved ejection fraction/ or heart failure with reduced ejection fraction/	283676	
3	heart failure.mp.	452584	
4	1 or 2 or 3	497666	1-3: different search terms for heart failure
5	trimetazidine.mp. or trimetazidine/ or vastarel/	2958	
6	Randomized controlled trial/	705832	
7	Controlled clinical trial/	465493	
8	random$.ti,ab.	1780539	
9	randomization/	93681	
10	intermethod comparison/	282332	
11	placebo.ti,ab.	339706	
12	(compare or compared or comparison).ti.	562645	
13	((evaluated or evaluate or evaluating or assessed or assess) and (compare or compared or comparing or comparison)).ab.	2484348	
14	(open adj label).ti,ab.	96408	
15	((double or single or doubly or singly) adj (blind or blinded or blindly)).ti,ab.	255617	
16	double blind procedure/	194291	
17	parallel group$1.ti,ab.	29248	
18	(crossover or cross over).ti,ab.	115857	
19	((assign$ or match or matched or allocation) adj5 (alternate or group$1 or intervention$1 or patient$1 or subject$1 or participant$1)).ti,ab.	377849	
20	(assigned or allocated).ti,ab.	445077	
21	(controlled adj7 (study or design or trial)).ti,ab.	405538	
22	(volunteer or volunteers).ti,ab.	266926	
23	human experiment/	572212	
24	trial.ti.	356654	
25	or/6-24	5738838	
26	(random$ adj sampl$ adj7 (cross section$ or questionnaire$1 or survey$ or database$1)).ti,ab. Not (comparative study/ or controlled study/ or randomi?ed controlled.ti,ab. Or randomly assigned.ti,ab.)	8952	

27	Cross-sectional study/ not (randomized controlled trial/ or controlled clinical study/ or controlled study/ or randomi?ed controlled.ti,ab. Or control group$1.ti,ab.)	307210	
28	(((case adj control$) and random$) not randomi?ed controlled).ti,ab.	19725	
29	(Systematic review not (trial or study)).ti.	207267	
30	(nonrandom$ not random$).ti,ab.	17725	
31	Random field$.ti,ab.	2689	
32	(random cluster adj3 sampl$).ti,ab.	1432	
33	(review.ab. and review.pt.) not trial.ti.	987216	
34	we searched.ab. and (review.ti. or review.pt.)	41519	
35	update review.ab.	122	
36	(databases adj4 searched).ab.	50194	
37	(rat or rats or mouse or mice or swine or porcine or murine or sheep or lambs or pigs or piglets or rabbit or rabbits or cat or cats or dog or dogs or cattle or bovine or monkey or monkeys or trout or marmoset$1).ti. and animal experiment/	1146615	
38	Animal experiment/ not (human experiment/ or human/)	2406754	
39	or/26-38	3944435	
40	25 not 39	5083872	6-40: Cochrane Embase RCT filter sensitivity-maximising version (2008 revision)(18)
41	4 and 5 and 40	170	Final dataset

Table A1-C

Search strategy for Cochrane CENTRAL

	Input	Hits	Comments
#1	MeSH descriptor: [Heart Failure] explode all trees	10316	
#2	cardiac NEAR/2 failure	2571	
#3	heart NEAR/2 decompensation	60	
#4	myocardial NEAR/2 failure	982	1-3: different search terms for heart failure
#5	#1 OR #2 OR #3 OR #4	12666	
#6	MeSH descriptor: [Trimetazidine] explode all tree	213	
#7	Trimetazidine OR vastarel	484	
#8	#6 OR #7	484	
#9	#5 AND #8	22	Final dataset

Table A1-D

Search strategy for www.clinicaltrials.gov

	Input	Hits	Comments
1	Heart failure AND trimetazidine	1	Final dataset

Table A2

Full-text papers assessed for eligibility. Studies that have not been included in previous systematic reviews are highlighted in yellow, studies that were excluded are highlighted in grey.

First author and year-of-publication	Titel	Reference	Final assessment
Al-Shawabkeh 2013	Trimetazide therapy in patients with dilated cardiomyopathy and congestive heart failure	Eur J Heart Fail Suppl 2013;12:S316	Conference abstract
An 2019	Effect of trimetazidine on elderly patients in heart failure vulnerable period	J Am Ger Soc 2019;67(Suppl 4):S598	Conference abstract
Bohdan 2022	Effects of trimetazidine in patients with severe chronic heart failure with reduced left ventricular ejection fraction: A prospective, randomized, open-label, cross-over study	Cardiol J 2022;29:627-36	Included in the meta-analysis
Bricaud 1990	Cardioprotective effect of trimetazidine in severe ischemic cardiomyopathy	Cardiovasc Drugs Ther 1990;4(Suppl 4):861-5	Included in the meta-analysis
Bubnova 2016	Efficacy of trimetazidine – an inhibitor of free fatty acids oxidation in the treatment of patients with stable angina pectoris and heart failure	Eur J Heart Fail 2016;18(Suppl 1):191	Conference abstract
Bubnova 2021	Efficacy of trimetazidine – an inhibitor of free fatty acids oxidation in the treatment of patients with stable angina pectoris and heart failure	Kardiologiia 2021;61:65-76	Included in the meta-analysis
Caminiti 2016	Effect of trimetazidine on exercise capacity and muscle strength in patients with HFrEF	Eur J Heart Fail 2016;18(Suppl 1):78	Conference abstract
Coats 2019	Effect of trimetazidine dihydrochloride therapy on exercise capacity in patients with nonobstructive hypertrophic cardiomyopathy: a randomized clinical trial	JAMA Cardiol 2019;4:230-5	Included in the meta-analysis
Dana 2009	Is trimetazidine, “on top of” standard treatment, useful in patients with preserved ejection fraction heart failure of ischaemic etiology?	Eur J Heart Fail 2009;2:ii621-2	Conference abstract
Fedorova 2004a	[Potentialities of cytoprotection in the treatment of chronic heart failure in patients with coronary heart disease]	Klin Med (Mosk) 2004;82:15-20.	Included in the meta-analysis
Fedorova 2004b	[Clinical and hemodynamic efficacy of selective beta-blocker bisoprolol and cytoprotector trimetazidine in the treatment of chronic cardiac failure in patients with ischemic heart disease]	Ter Arkh 2004;76:62-8	Double publication (Fedorova 2004a)
Fragasso 2006a	A randomized clinical trial of trimetazidine, a partial free fatty acid oxidation inhibitor, in patients with heart failure	J Am Coll Cardiol 2006;48:992-8	Included in the meta-analysis

Fragasso 2006b	Effects of metabolic modulation by trimetazidine on left ventricular function and phosphocreatine/adenosine triphosphate ratio in patients with heart failure	Eur Heart J 2006;27:942-8	Included in the meta-analysis
Fragasso 2011	Effect of partial inhibition of fatty acid oxidation by trimetazidine on whole body energy metabolism in patients with chronic heart failure	Heart 2011;97:1495-1500	Included in the meta-analysis
Isser 2013	A randomized controlled trial to evaluate the role of trimetazidine in non-ischemic heart failure	Indian Heart J 2013;1:S65	Conference abstract
Jatain 2016	Metabolic manipulation in dilated cardiomyopathy: Assessing the role of trimetazidine	Indian Heart J 2016;68:803-8	Included in the meta-analysis
Lee 2018	Trimetazidine as a long-term add-on therapy in ischemic cardiomyopathy did not reduce cardiac mortality and rehospitalization	Circulation 2018;138(Suppl_1):A10920	Conference abstract
Lopatin 2012	Long-term trimetazidine modified release therapy improves prognosis in post-myocardial infarction patients with angina pectoris and heart failure	Eur Heart J 2012;1(Suppl):346-7	Conference abstract
Marazzi 2016	Effect of long-term treatment with trimetazidine on left ventricular systolic function, on quality of life and capacity in patients with chronic heart failure	Eur J Heart Fail 2016;18(S1):51	Conference abstract
Momen 2016	Effects of sustained-release trimetazidine on chronically dysfunctional myocardium of ischemic dilated cardiomyopathy: Six months follow-up result	Indian Heart J 2016; 68:809-15	Included in the meta-analysis
Morozova 2011	[Clinico-economical aspects of application of trimetazidine MB in patients with chronic heart failure and cardiac rhythm disturbances]	Kardiologiia 2011;51:35-41	Included in the meta-analysis
Di Napoli 2007a	Trimetazidine and reduction in mortality and hospitalization in patients with ischemic dilated cardiomyopathy: a post hoc analysis of the Villa Pini d'Abruzzo Trimetazidine Trial	J Cardiovasc Pharmacol 2007;50:585-9	Included in the meta-analysis
Pagorek 2013	Trimetazidine improves exercise tolerance and left ventricular function in patients with contemporary treatment of chronic ischemic heart failure	Eur Heart J 2013;1(Suppl):162	Conference abstract
Perseghin 2010	Effect of partial inhibition of fatty-acid oxidation by trimetazidine on whole body energy metabolism in diabetic and non-diabetic patients with chronic heart failure	Diabetes 2010;59(Suppl 1):2126-PO	Conference abstract
Qin 2020	Efficacy of valsartan combined with trimetazidine in the treatment of chronic heart failure and effects on cardiac function	Int J Clin Exp Med 2020;13:155-63	Included in the meta-analysis
Salerno 2010	Decreased rate of energy expenditure induced by trimetazidine, a partial inhibition of fatty acid oxidation, in patients with chronic heart failure	Eur Heart J 2010;1(Suppl):851	Conference abstract

Sedova 2010	[A clinical experience of taurine and trimetazidine use in premenopausal women with chronic heart failure]	Kardiologiia 2010;50:62-3	Included in the meta-analysis
Sabrov 2003	Effect of trimetazidine and perindopril on myocardial function and metabolism in patients with low cardiac output during postinfarction period	Ter Arkh 2003;75:77-81	No outcome data of interest reported
Sisakian 2007	The effect of trimetazidine on left ventricular systolic function and physical tolerance in patients with ischaemic cardiomyopathy	Acta Cardiologica 2007;62:493-9	Included in the meta-analysis
Sitnikova 2015	[Management of angina attacks in heart failure due to noncoronary heart disease: Trimetazidine usage]	Russ J Cardiol 2015;127:81-5	Included in the meta-analysis
Vasiuk 2007	[Comparative trial of efficacy of trimethasidine MB and 3-(2,2,2-trimethylhydrasine) propionate dihydrate in chronic heart failure]	Ter Arkh 2007;79:51-8.	Included in the meta-analysis
Wang 2020	Shenmai injection improves energy metabolism in patients with heart failure: a randomized controlled trial	Front Pharmacol 2020;11:459	Included in the meta-analysis
Wang 2012	Trimetazidine on cardiac function of ischaemic cardiomyopathy clinical observation	Heart 2012;2:E173	Conference abstract
Winter 2014	Effects of trimetazidine in nonischemic heart failure: a randomized study	J Card Fail 2014;20:149-154	Included in the meta-analysis
Yuanhong 2017	The cardioprotective effects of trimetazidine in dilated cardiomyopathy patients with left bundle branch block	J Am Coll Cardiol 2017;70(Suppl 1): C128-9	Conference abstract
Zhang 2017	Efficacy and safety of metoprolol plus trimetazidine in treating coronary heart failure	Biomed Res 2017;28:4549-52	Included in the meta-analysis
Brottier 1990	Therapeutic value of a cardioprotective agent in patients with severe ischaemic cardiomyopathy	Eur Heart J 190;11:207-12	Identified by hand search
Van de Bovenkamp 2023	Trimetazidine in heart failure with preserved ejection fraction: a randomised controlled cross-over trial	ESC Heart Fail 2023;	Identified by hand search
Cera 2010	Beneficial electrophysiological effects of trimetazidine in patients with postischemic chronic heart failure	J Cardiovasc Pharmacol Ther 2010;15:24-30	Identified by hand search
Gunes 2009	Improved left and right ventricular functions with trimetazidine in patients with heart failure: A tissue Doppler study	Heart Vessels 2009;24:277-82	Identified by hand search
El-Kady 2005	Effects of trimetazidine on myocardial perfusion and the contractile response of chronically dysfunctional myocardium in ischemic cardiomyopathy: A 24-month study	Am J Cardiovasc Drugs 2005;5:271-8	Identified by hand search

Di Napoli 2007b	Beneficial effects of trimetazidine treatment on exercise tolerance and B-type natriuretic peptide and troponin T plasma levels in patients with stable ischemic cardiomyopathy	Am Heart J 2007;154:602.e1-5	Identified by hand search
Tuunanen 2008	Trimetazidine, a metabolic modulator, has cardiac and extracardiac benefits in idiopathic dilated cardiomyopathy	Circulation 2008;118:1250-8	Identified by hand search
Vitale 2004	Trimetazidine improves left ventricular function and quality of life in elderly patients with coronary artery disease	Eur Heart J 2004;25:1814-21	Identified by hand search

Table A3

Study					Patient[a]						Trimetazidine				Control			Reported outcomes of interest				
First author	Year	Country	Language	N (I/C)	Incl. criteria	LVEF (%)	Age (yrs)	Sex (%F)	BMI (kg/m^2)	DM (%)	Daily dose (mg)	F-U (m)	AE	ED	Placebo/ SoC (HF-therapy)	AE	ED	M	H	FC	6	QoL
Bohdan(32)	2022	Poland	English	22/23	HFrEF/iCMP	24/23	59/57	0/9	28/29	14/35	2x35	6	1*	0	Open-label (A, B, M)	NR*	NR*	Y	Y	Y	Y	MacNew
vd Bovenkamp(22)	2023	Neth.	English	25[b]	HFpEF	58	66	60	30	16	2x35	4	33	0	Placebo (A, B, M)	21	0	Y	-	Y	Y	KCCQ
Bricaud(33)	1990	France	English	9/11	HFrEF/iCMP	29/23	57/62	0/9	-	-	3x20	6	0	0	Placebo (NR)	2	0	Y	-	Y	-	-
Brottier(34)	1990	France	English	9/11	HFrEF/iCMP	29/23	57/62	0/9	-	-	3x20	6	1	0	Placebo (NR)	3	0	-	Y	Y	-	-
Bubnova(23)	2021	Russia	Russian	691/115	iCMP	45/44	57/57	40/38	29/29	-	-	12	18	NR	Open-label (A, B, M)	59	NR	-	Y	Y	-	-
Cera(35)	2010	Italy	English	17/13	HFrEF/iCMP	38/33	65/70	12/15	-	41/31	3x20	6	NR	0	Open-label (A, B)	NR	0	-	-	Y	-	-
Coats(21)	2019	UK	English	27/24	HCM/n-iCMP	63/60	49/51	33/25	29/28	-	3x20	3	0	0	Placebo (NR)	1	1	-	-	-	Y	MLWHFQ
Fedorova(24)	2004	Russian	Russian	20/24	HFrEF/iCMP	29/29	64/62	-	-	-	3x20	6	2	0	Open-label (A, B)	3	0	Y	-	Y	-	MLWHFQ
Fragasso(36)	2006a	Italy	English	28/27	HFrEF	34/36	64/66	11/7	-	4/11	3x20	12	1	0	Open-label (A, B)	2	0	Y	Y	Y	-	VAS
Fragasso(6)	2006b	Italy	English	12[b]	HFrEF	33	66	8	-	-	3x20	3	0	0	Open-label (A, B)	0	0	-	-	Y	-	VAS
Fragasso(37)	2011	Italy	English	25/19	HFrEF	35/35	70/69	12/16	28/30	36/32	3x20	3	0	0	Open-label (A, B)	1	0	-	-	Y	-	VAS
Gunes(38)	2009	Turkey	English	51/36	HFrEF	33/31	69/67	27/42	26/25	27/31	3x20	3	NR	NR	Open-label (A, B, M)	NR	NR	-	-	Y	-	-
Jatain(39)	2016	India	English	50/50	HFrEF/n-iCMP	27/28	47/48	26/24	-	20/20	3x20	6	0	0	Open-label (A, B)	0	0	Y	Y	Y	Y	-
El-Kady(40)	2005	Egypt	English	100/100	HFrEF/iCMP	36/37	53/53	14/22	21/19	37/30	3x20	24	0	0	Open-label (A)	0	0	Y	-	-	-	-
Momen(41)	2016	Banglad.	English	55/53	HFrEF/iCMP	33/33	58/59	18/23	-	47/51	3x20	6	NR*	0	Placebo (A, B, M)	NR*	0	-	Y	Y	-	-
Morozova(25)	2011	Russia	Russian	40/42	HFrEF	35/37	63/61	23/14	-	-	2x35	3,5	NR	NR	Open-label (A, B)	NR	NR	-	-	Y	-	-
Di Napoli(42)	2007a	Italy	English	30/31	HFrEF/iCMP	30/31	67/69	43/42	-	33/35	3x20	30	0	0	Open-label (A, B, M)	0	0	Y	Y	Y	Y	-
Di Napoli(43)	2007b	Italy	English	25/25	HFrEF/iCMP	30/31	64/63	40/28	-	33/35	3x20	6	0	0	Placebo (A, B, M)	0	0	-	-	Y	Y	-

Qin(44)	2020	China	English	41/41	n-iCMP	42/41	71/72	39/32	26/26	28/20	3x20	3	7	0	Open-label (A)	12	0	-	-	Y	-	SF-36
Sedova(26)	2010	Russia	Russian	35/33	n-iCMP	-	-	100/100	-	20/27	2x35	1	0	0	Open-label (A, B)	0	0	-	-	Y	-	-
Sisakian(45)	2007	Armenia	English	42/40	HFrEF/iCMP	35/32	64/66	12/18	-	-	2x35	3	1	0	Open-label (A, B)	0	0	-	-	Y	Y	-
Sitnikova(27)	2015	Russia	Russian	20/15	HFrEF/n-iCMP	35/34	47/49	25/20	27/26	0/0	2x35	12	NR	0	Open-label (A, B, M)	NR	0	-	Y	-	-	MLWHFQ
Tuunanen(46)	2008	Finland	English	12/7	HFrEF/n-iCMP	31/38	59/57	17/29	27/30	-	2x35	3	2	0	Open-label (A, B)	2	0	-	Y	-	-	-
Vasiuk(28)	2007	Russia	Russian	28/12	HFrEF/iCMP	40/41	63/63	38/40	-	0/0	2x35	6	NR	NR	Open-label (NR)	NR	NR	Y	Y	Y	-	-
Vitale(30)	2004	Italy	English	23/24	HFrEF/iCMP	30/33	77/78	0/30	27/27	33/25	3x20	6	1	0	Placebo (A)	2	0	-	Y	Y	-	VAS
Wang(47)	2020	China	English	39/40	All HF	-	70/72	23/23	-	13/3	2x35	0,25	NR	0	Placebo (A, B, M)	NR	0	-	-	Y	Y	-
Winter(48)	2014	Chili	English	30/30	HFrEF/n-iCMP	29/29	53/57	22/8	24/24	-	2x35	6	12	0	Placebo (A, B, M)	5	0	Y	Y	Y	Y	MLWHFQ
Zhang(29)	2017	China	English	100/100	HFrEF/iCMP	36/36	65/61	42/38	21/21	30/21	3x20	1	NR*	0	Open-label (NR)	NR*	0	Y	-	-	Y	-

Key features on study design and general characteristics on study population of the included studies. [a]: For clarity, only mean LVEF, age, etc. of the intervention/control group are listed; [b]: cross-over study.

Abbreviations: Year, year-of-publication; Language, language used in the report; N (I/C), number of patients in the intervention/control group; Incl. criteria, main inclusions criteria; NYHA, New York Heart Association functional class; LVEF, left ventricular ejection fraction; %F, percentage females; BMI, body mass index; DM, (type 2) diabetes mellitus; F-U (m), follow-up (months); SoC, standard-of-care (open-label); AE, adverse event; ED, early discontinuation; A, angiotensin converting enzyme inhibitor or angiotensin receptor blocker; B, beta-blocker; M, mineralocorticoid antagonist; NR, not reported; *, adverse events reported, unclear in which study group; M, mortality; H, (heart failure) hospitalisation; FC, NYHA functional class, 6, 6-minute walk distance, QoL, quality-of-life (specific questionnaire listed); Neth., The Netherlands; HFrEF/HFpEF, heart failure with (mildly) reduced (LVEF<50%) or preserved ejection fraction (LVEF≥50%); KCCQ, Kansas City Cardiomyopathy Questionnaire; iCMP/n-iCMP, ischaemic /non-ischaemic cardiomyopathy; HCM, hypertrophic cardiomyopathy; all HF, included if recent heart failure hospitalisation (irrespective of HF aetiology, LVEF); Y, yes, outcome of interest reported; UK, United Kingdom; MLWHFQ, Minnesota Living With Heart Failure Questionnaire; VAS, visual analogue scale; Banglad., Bangladesh.

Table A4

	vd Bovenkamp 2023(22)	Gao 2011(10)	Zhang 2012(11)	Zhou 2014(12)	Grajek 2015(13)	Y.Zhao 2016(14)	C.Zhao 2021(15)
CV-mortality	11 (events: 90)	4 (events: 66)	3 (events: 56)	3 (events: 56)	3 (events: 56)	-	-
HF-hospitalisation	12 (events: 229)	3 (N=93)	4 (N=24)	4 (N=24)	-	-	-
NYHA functional class	23 (N=2084)	7 (N=426)	7 (N=402)	8 (N=449)	-	-	-
6MWD	10 (N=699)	-	-	-	-	3 (N=218)	3 (N=211)
QoL	8 (N=537)	-	-	-	-	-	4 (N=213)
Total number of studies included	28	9	9	11	3	3	5

Comparison of systematic reviews on trimetazidine in HF and the number of studies included per clinical endpoint and in total. Comparison of systematic reviews on trimetazidine in HF. The number of studies that are included in the meta-analysis, with the total events of number or patients in brackets (indicator of the statistical power), are specified per clinical endpoint Abbreviations: CV, cardiovascular; HF, heart failure; NYHA, New York Heart Association; 6MWD, 6-minute walk distance; QoL, quality-of-life.

Supplemental figures:

Figure A1-A

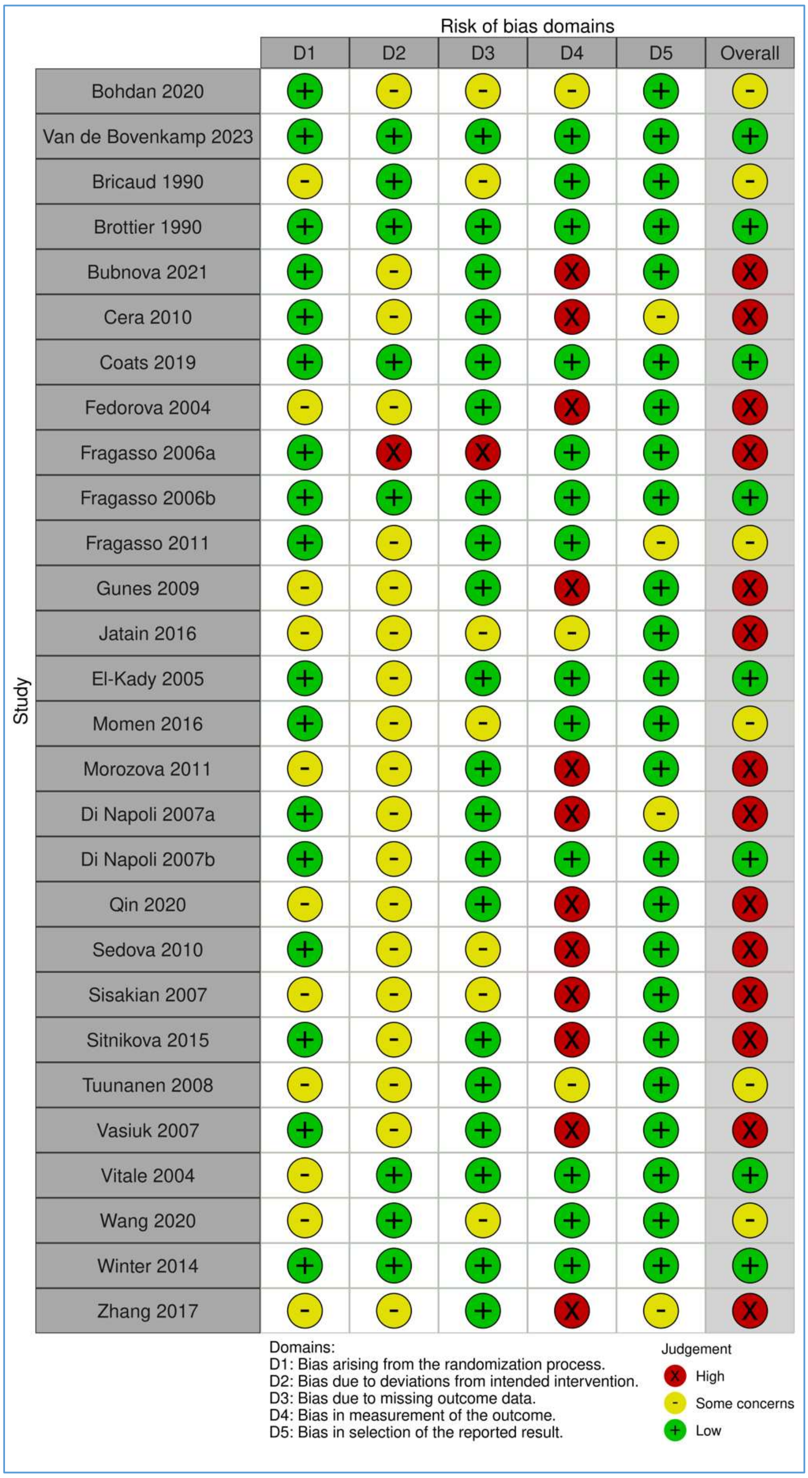

Traffic light plot: domain-level judgement of risk-of-bias according to risk-of-bias 2 tool.

Figure A1-B

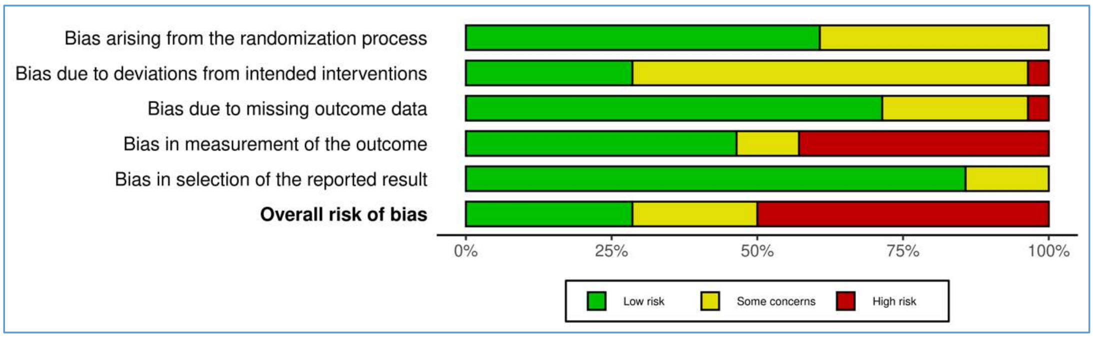

Summary plot: weighted bar plots of the distribution of risk-of-bias judgements within each bias domain of the 28 included studies in the meta-analysis.

Figure A2-A

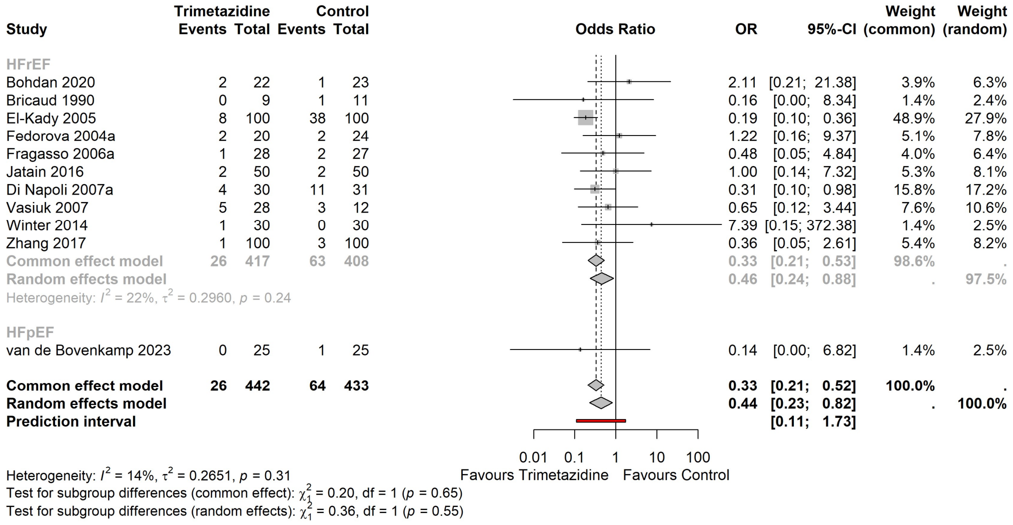

Forest plot: the effect of trimetazidine in HF on (cardiovascular) mortality (11 studies), organized by HFrEF vs. HFpEF.

Figure A2-B

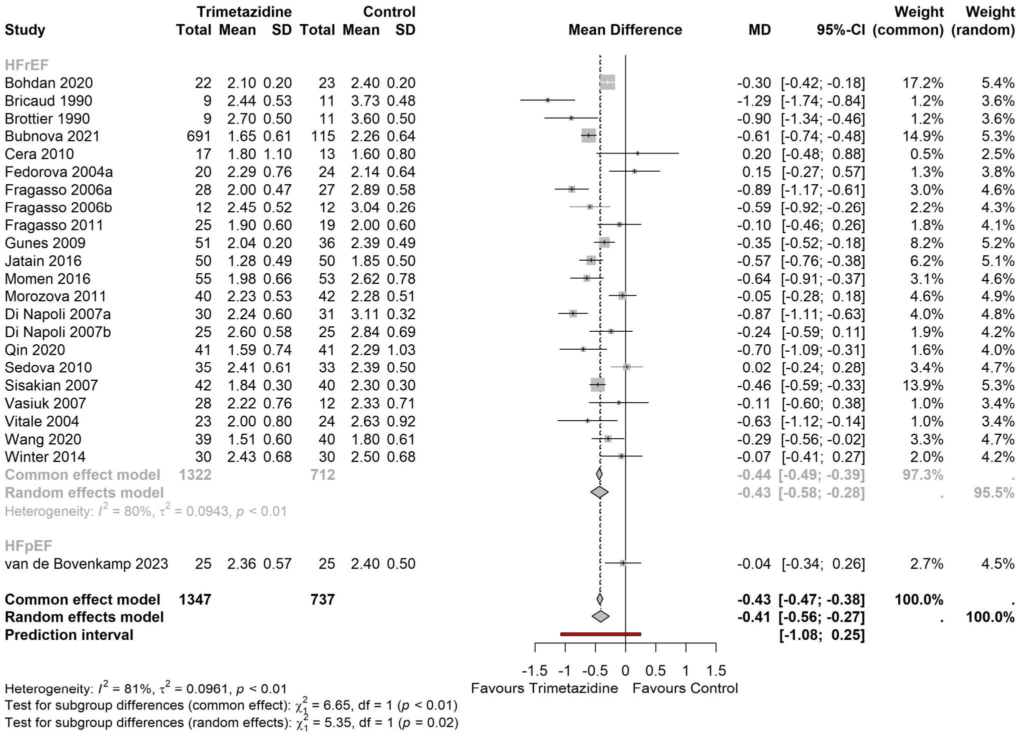

Forest plot: the effect of trimetazidine in HF on NYHA functional class (23 studies), organized by HFrEF vs. HFpEF (significant interaction, less effect in HFpEF).

Figure A2-C

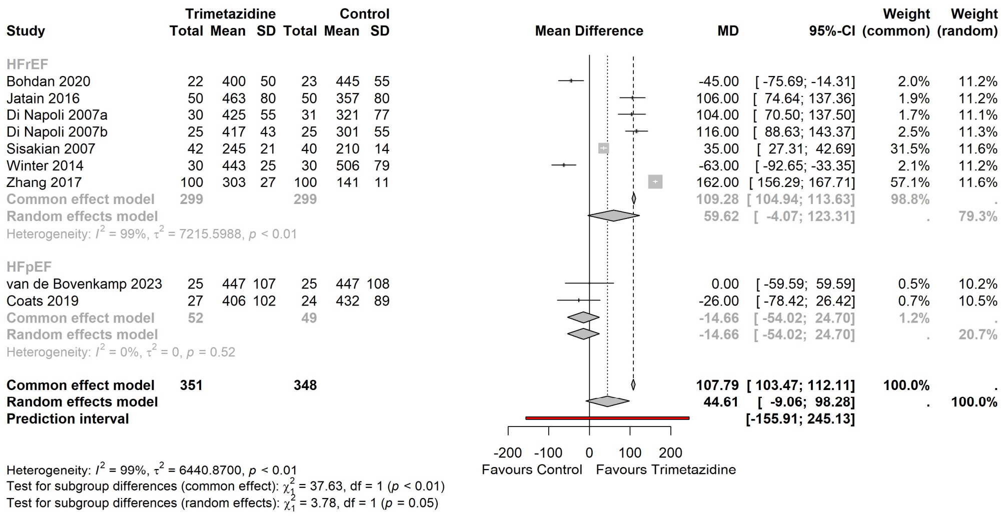

Forest plot: the effect of trimetazidine in HF on 6-minute walk distance (9 studies), organized by HFrEF vs. HFpEF (significant interaction, less effect in HFpEF).

Figure A2-D

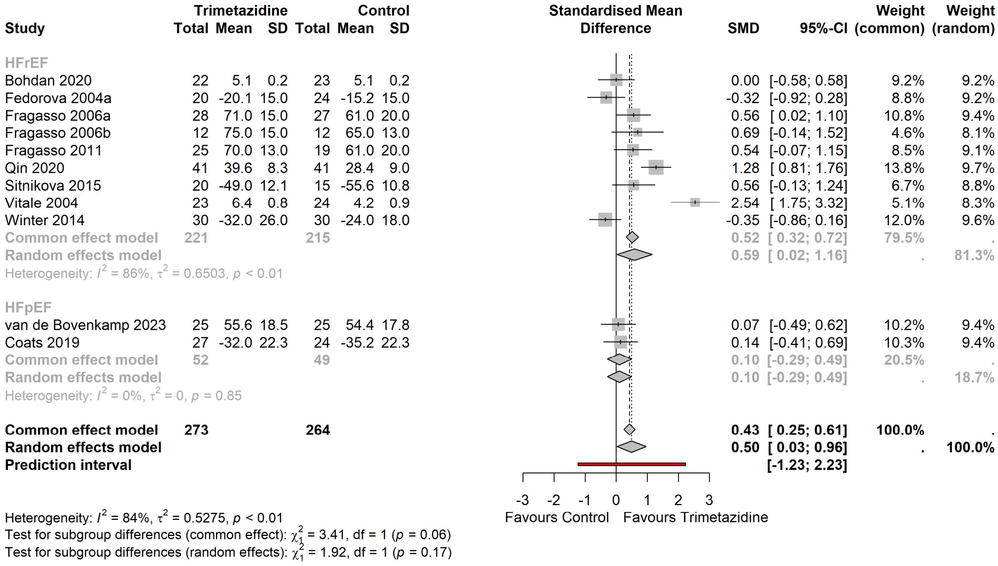

Forest plot: the effect of trimetazidine in HF on quality-of-life (11 studies), organized by HFpEF vs. HFrEF (borderline significant interaction, less effect in HFpEF).

Figure A3-A

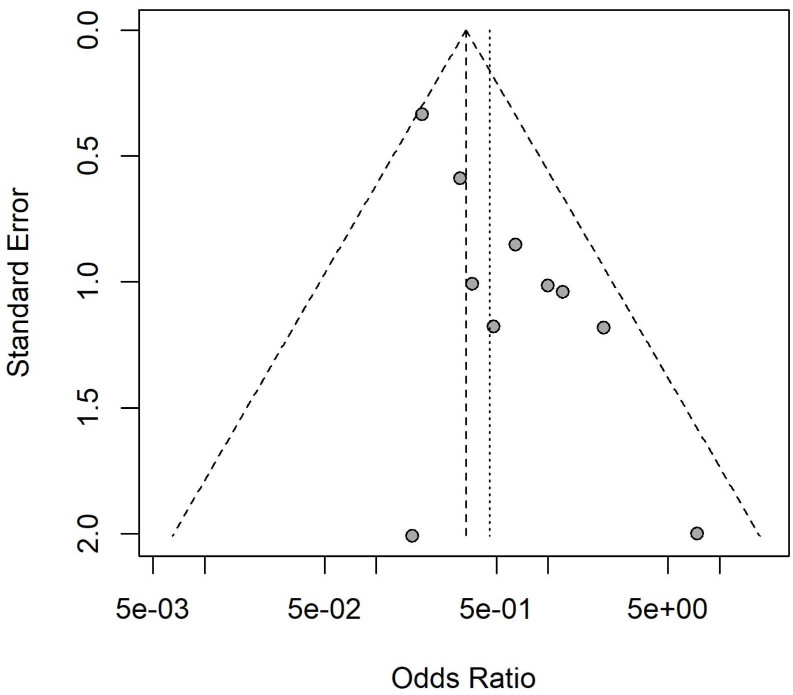

Funnel plot of trimetazidine in HFrEF on (cardiovascular) mortality. Egger's test for asymmetry: p=0.006.

Figure A3-B

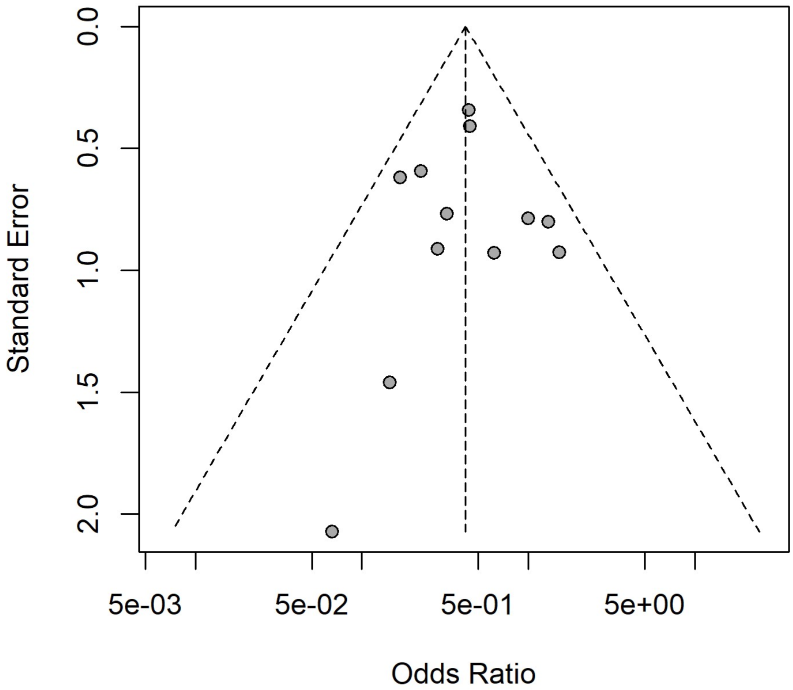

Funnel plot of trimetazidine in HFrEF on HF-hospitalisations. Egger's test: p=0.90.

Figure A3-C

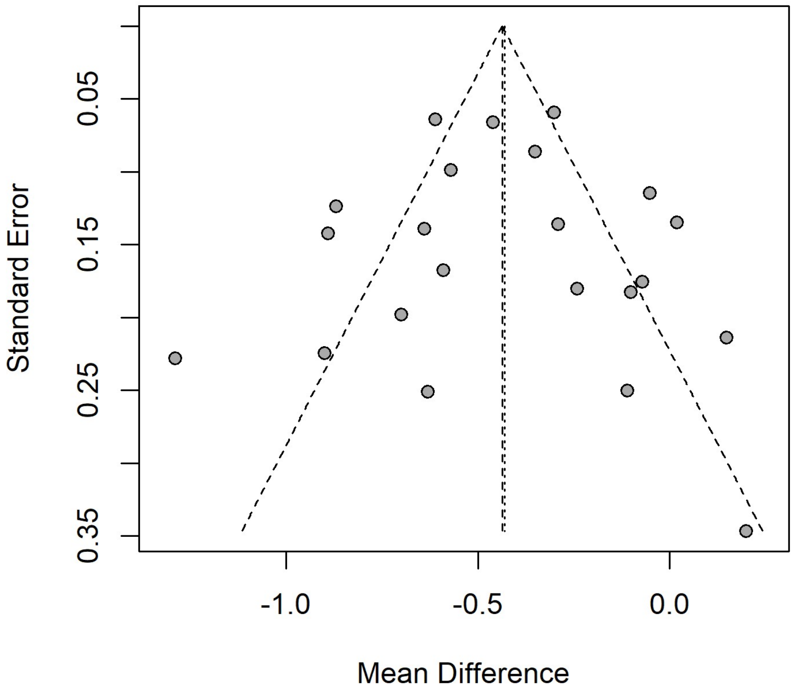

Funnel plot of trimetazidine in HFrEF on NYHA functional class. Egger's test: p=0.90.

Figure A3-D

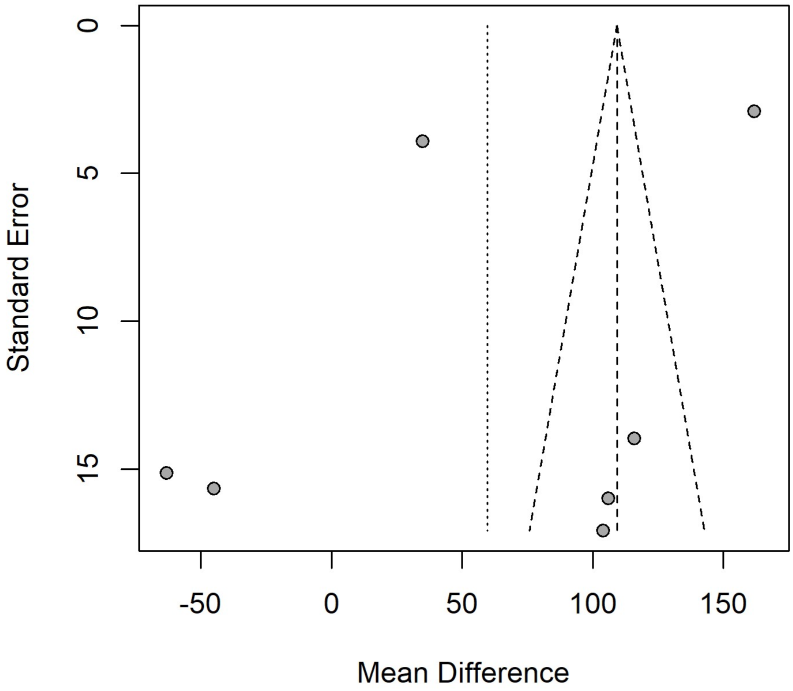

Funnel plot of trimetazidine in HFrEF on 6-minute walk distance. Egger's test: p=0.30.

Figure A3-E

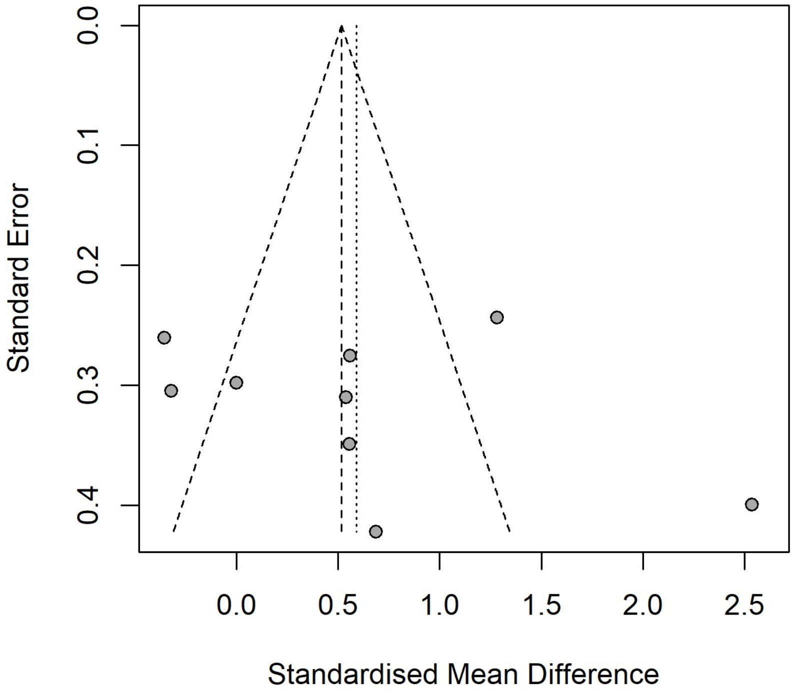

Funnel plot of trimetazidine in HFrEF on quality-of-life. Egger's test: p=0.40.
